# Investigating the Urinary Metabolome in the First Year of Life and Its Association with Later Diagnosis of Autism Spectrum Disorder or Non-Typical Neurodevelopment in the MARBLES Study

**DOI:** 10.3390/ijms24119454

**Published:** 2023-05-29

**Authors:** Jennie Sotelo-Orozco, Rebecca J. Schmidt, Carolyn M. Slupsky, Irva Hertz-Picciotto

**Affiliations:** 1 Department of Public Health Sciences, School of Medicine, University of California Davis, Davis, CA 95616, USA; rjschmidt@ucdavis.edu (R.J.S.); iher@ucdavis.edu (I.H.-P.); 2 Medical Investigation of Neurodevelopmental Disorders (MIND) Institute, School of Medicine, University of California Davis, Sacramento, CA 95817, USA; 3 Department of Nutrition, University of California, Davis, CA 95616, USA; cslupsky@ucdavis.edu; 4 Department of Food Science and Technology, University of California, Davis, CA 95616, USA

**Keywords:** autism spectrum disorder, children, biochemical markers, metabolome, NMR, serine, hippurate, tryptophan

## Abstract

Developmental disabilities are often associated with alterations in metabolism. However, it remains unknown how early these metabolic issues may arise. This study included a subset of children from the Markers of Autism Risks in Babies—Learning Early Signs (MARBLES) prospective cohort study. In this analysis, 109 urine samples collected at 3, 6, and/or 12 months of age from 70 children with a family history of ASD who went on to develop autism spectrum disorder (ASD *n* = 17), non-typical development (Non-TD *n* = 11), or typical development (TD *n* = 42) were investigated by nuclear magnetic resonance (NMR) spectroscopy to measure urinary metabolites. Multivariate principal component analysis and a generalized estimating equation were performed with the objective of exploring the associations between urinary metabolite levels in the first year of life and later adverse neurodevelopment. We found that children who were later diagnosed with ASD tended to have decreased urinary dimethylamine, guanidoacetate, hippurate, and serine, while children who were later diagnosed with Non-TD tended to have elevated urinary ethanolamine and hypoxanthine but lower methionine and homovanillate. Children later diagnosed with ASD or Non-TD both tended to have decreased urinary 3-aminoisobutyrate. Our results suggest subtle alterations in one-carbon metabolism, gut-microbial co-metabolism, and neurotransmitter precursors observed in the first year of life may be associated with later adverse neurodevelopment.

## 1. Introduction

Rapid periods of neurodevelopment initiate prenatally and continue through early infancy [1]. Intricate interactions between genetics, nutrition, and environmental influences occurring during these critical periods of development have been associated with developmental disabilities [2,3]. Developmental disabilities are estimated to affect about one in six (17%) children aged 3–17 in the United States [4]. These include autism spectrum disorder (ASD), schizophrenia, learning disability, cerebral palsy, and language and speech disorders. Moreover, although developmental disabilities affect learning, language, and behavior, an increasing body of evidence supports the view that developmental disabilities are often accompanied by disturbances in metabolism which affect various systems involved in complex and highly interdependent ways [5].

Metabolomics analysis offers unique insight into perturbances of metabolism as metabolites are products and intermediate molecules of metabolic pathways occurring throughout a biological system. Additionally, alterations at the metabolome level reflect disturbances in the genomics, transcriptomics, and proteomics cascade, and bridge the gap between the genome and phenotype. Ongoing studies are actively seeking to understand how complex interactions between genes, environment, microbiome, and metabolome affect autism [6], as numerous abnormal metabolic pathways have previously been found in the urine and blood of children with ASD [7]. In our previous work, we conducted a plasma metabolomics investigation in 3- to 5-year-old children with ASD (*n* = 167), Down syndrome (DS, *n* = 31), and idiopathic developmental disabilities (i-DD, *n* = 51) compared to typically developing (TD, *n* = 193) children [8]. Despite the varied origins of these developmental disabilities, we found similar perturbations in one-carbon-related metabolic pathways among ASD cases and DS children. Additionally, similarities were also found among DS and i-DD cases in the tricarboxylic acid cycle (TCA), with similar trends observed among ASD cases. These findings contribute to the growing evidence of metabolic disturbances seen among children with ASD in particular metabolic pathways such as one-carbon-related metabolism, which has repeatedly been implicated in autism [9,10,11,12,13,14]. Several other metabolic pathways have also repeatedly been associated with ASD, for example, alterations in amino acids that function as neurotransmitter precursors including tryptophan and glutamine [12,15,16,17,18]. Alterations in gut-microbial-derived metabolites, such as hippurate and p-cresol, have also repeatedly been implicated in ASD, suggesting alterations in the microbiota–gut–brain axis [12,19,20,21,22,23]. However, it remains unknown how early metabolic abnormalities may arise in ASD and other developmental disabilities. Investigating early metabolic trajectories—even before neurodevelopmental diagnoses are made—may help guide strategies that directly target metabolism and possibly reduce ASD incidence/severity.

The objective of the present study was to investigate early alterations in key metabolic pathways by investigating differences in the urinary metabolome of infants at 3, 6, and 12 months of age in children who were later diagnosed with ASD, or non-typical development (Non-TD), compared to neurotypical controls (TD). By investigating ASD, and Non-TD, compared to TD controls, we can better characterize metabolic differences and similarities associated with these neurodevelopmental disabilities. Building off the existing literature and our previous analysis, we hypothesized a priori that alterations in one-carbon-metabolism-related metabolites (serine, betaine, N,N-dimethylglycine, glycine, choline, methionine, 2-aminobutyrate, and 2-hydroxybutyrate) would differ among children with ASD compared to TD controls. Additionally, we hypothesized some overlap between Non-TD and ASD children including TCA cycle metabolites (2-oxoglutarate, cis-aconitate, citrate, fumarate, succinate) though expected Non-TD cases to also have unique metabolic differences distinguishing them from ASD and TD. Differences were also expected in urinary-specific metabolites produced by the gut microbiome (e.g., hippurate) among ASD children compared to Non-TD and TD. Overall, our goal was to investigate the early urinary metabolome differences associated with later adverse neurodevelopment, focusing on key metabolic pathways repeatedly implicated in ASD.

## 2. Results

**Figure 1:**
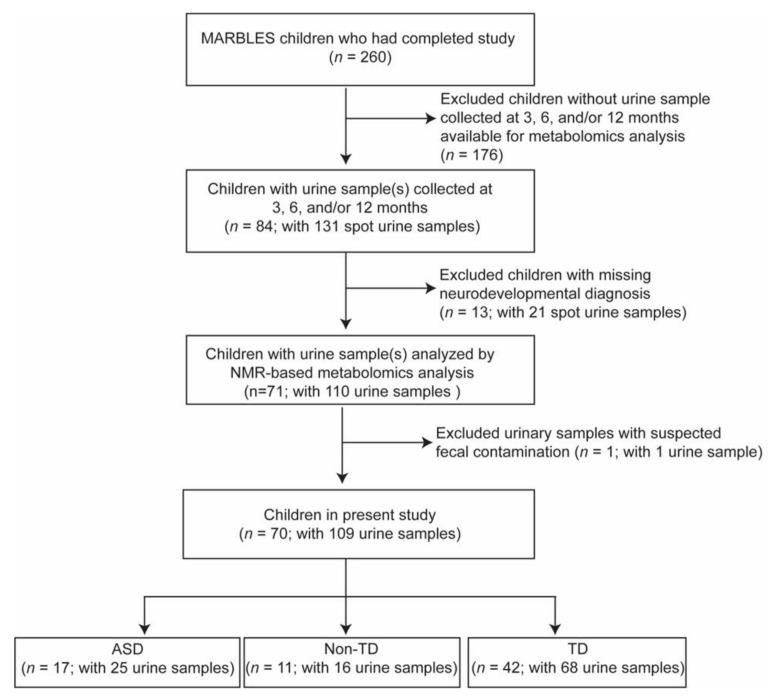
Flow chart of study population selection.

Table 1 shows the study population demographics for this subset of MARBLES participants. More ASD cases were males compared to females, as expected due to the 4:1 male-to-female ratio seen in autism across the literature, though the proportion of males to females was about equal among those with TD and Non-TD in our study population. In our study population, children with non-TD tended to be younger at the age of introduction to complementary foods/solids compared to ASD and TD cases, but this was not statistically significant. Breastfeeding duration was similar across diagnoses, with most children still receiving some/any breastmilk beyond 12 months of age; there was no difference based on diagnosis, although TD children tended to receive breastmilk longer compared to ASD and Non-TD cases. Racial/ethnic proportions were also similar between ASD, Non-TD, and TD groups, with about equal frequencies of White, Hispanic, or other ethnicities. Socioeconomic factors were also similar across groups, with most participants’ parents having used private insurance to pay for the child’s delivery, although a greater proportion of mothers of children with ASD tended to be renters compared to mothers of children with non-TD or TD, but this was not statistically significant. Similarly, maternal level of education did not differ significantly across groups; however, mothers of children with Non-TD tended to be less educated than mothers of children with TD or ASD. Interestingly, most mothers in our study population tended to be older with no significant difference observed between groups. Figure 1 illustrates the flow chart of the sample selection for the study.

Metabolomics analysis was performed on children with ASD (*n* = 17), Non-TD (*n* = 11), and TD (*n* = 42) to investigate urinary metabolite differences at 3, 6, and 12 months of age. A total of 83 metabolites of diverse chemical classes were identified in urine samples and included in an overall analysis. These included amino acids and their metabolites (1-methylnicotinamide, 2-oxobutyrate, 3-aminoisobutyrate, 3-hydroxyisovalerate, 3-methylhistidine, 4-aminobutyrate, asparagine, carnosine, creatine, creatinine, guanidoacetate, histidine, lysine, proline, taurine, threonine, and urocanate), branched-chain amino acids (BCAA) and their metabolites (3-hydroxy-3-methylglutarate, 3-hydroxyisobutyrate, isoleucine, leucine, and valine), glycolysis-related metabolites (lactate, pyruvate, and alanine), bacterial co-metabolites (1,2-propanediol, dimethyl sulfone, hippurate, trimethylamine, and trimethylamine-n-oxide), ketone bodies (3-hydroxybutyrate, acetoacetate, and acetone), lipid metabolism (carnitine and o-acetylcarnitine), short-chain fatty acids (acetate, butyrate, and propionate), one-carbon metabolism (choline, serine, betaine, N,N-dimethylglycine, glycine, methionine, 2-aminobutyrate, and 2-hydroxybutyrate), neurotransmitter precursors (phenylalanine, tyrosine, glutamate, glutamine, tryptophan (and its metabolite 3-indoxylsulfate)), sugars and their derivatives (fucose, galactose, gluconate, glucose, lactose, mannitol, and myo-inositol), tricarboxylic acid cycle (TCA) metabolites (2-oxoglutarate, cis-aconitate, citrate, fumarate, and succinate), and others (2-hydroxyisobutyrate, 4-hydroxyphenylacetate, adipate, ascorbate, dimethylamine, ethanolamine, ethylmalonate, formate, glycolate, homovanillate, hypoxanthine, methanol, methylguanidine, pantothenate, quinolinate, trigonelline, uracil, urea, and xanthosine).

**Figure 2:**
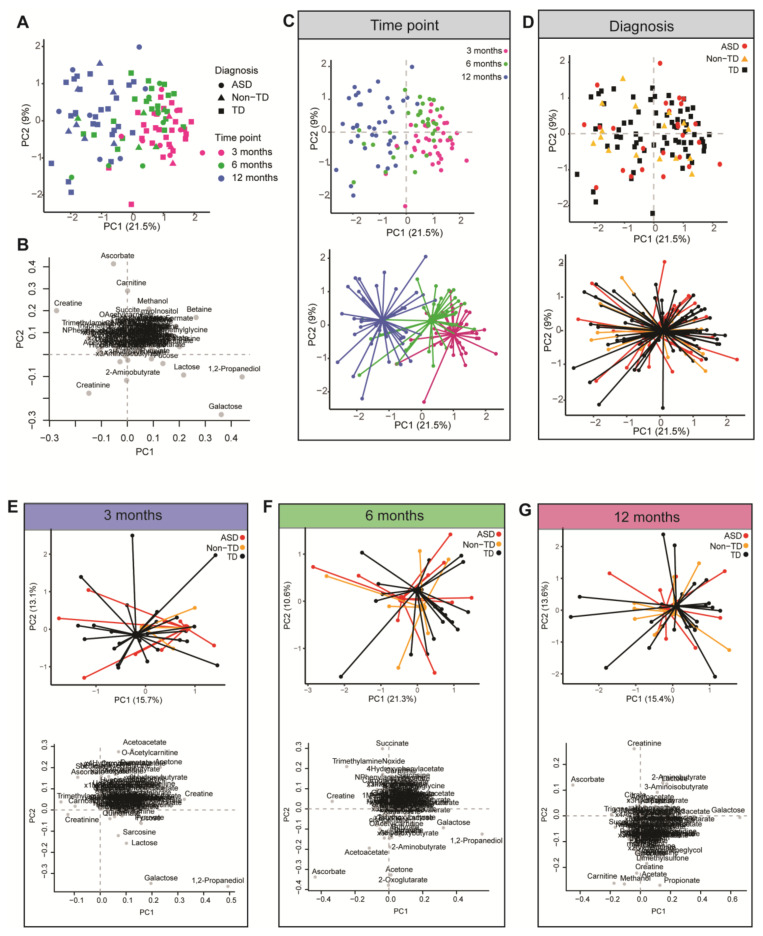
A comparison of the urinary metabolic profile of children with ASD, Non-TD, and TD at 3, 6, and 12 months of age based on PCA analysis. The shapes and colors indicate neurodevelopmental diagnosis and time points according to the legend. (**A**) PCA plot of participants. On the x-axis, PC1 accounts for 21.5% of the variation, and on the y-axis, PC2 accounts for 9% of the variation. (**B**) Corresponding loadings plot for PCA plot. The loadings plot indicates the contribution of the measured metabolites to the principal components. (**C**) Same as in A; however, PCA (top) and PCA centroid (bottom) plots are color-coded based on time point with clear differences in the urinary metabolome based on time point (3, 6, and 12 months of age) along with principal component 1 (PC1). (**D**) Same PCA plot as shown in A; however, PCA (top) and PCA centroid (bottom) plots are color-coded by diagnosis showing substantial overlap regardless of neurodevelopmental diagnosis. PCA and loadings plot for each time point: (**E**) 3 months, (**F**) 6 months, and (**G**) 12 months.

Multivariate PCA analysis was used to investigate inherent patterns in the metabolomic profiles (Figure 2). On the scores plot, each point represents a sample, and the loadings plot indicates the contribution of the measured metabolites to the principal components. Principal component 1 (PC1) accounted for 21.5% of the variation, and PC2 accounted for 9% of the variation on the scores plot. Clear differences in the urinary metabolic profile were observed based on time point, with tighter clustering observed at 3 months and dispersing at 12 months. However, there was no distinguishable cluster based on later neurodevelopmental diagnosis, indicating the urinary metabolome in the first year of life did not clearly distinguish metabolic profile based on a later neurodevelopmental diagnosis.

**Figure 3:**
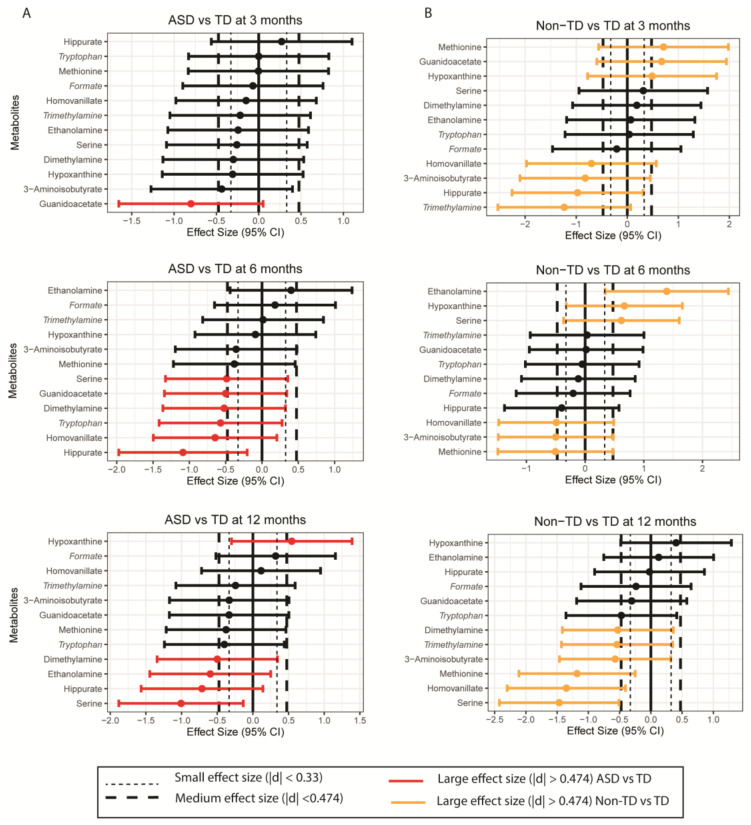
Effect size differences (95% CI) for metabolites with *p*-values (*p* < 0.05, unadjusted) and those trending toward statistical significance (*p* < 0.09, in italics) based on GEE results among (**A**) ASD vs. TD and (**B**) Non-TD vs. TD at each time point (3, 6 or 12 months).

Subsequently, GEE analysis was used to evaluate changes in the urinary metabolome in association with neurodevelopmental outcome (with TD as reference) while controlling for the child’s sex, age of introduction to solid foods, race and ethnicity, and parental homeownership (Table 2). In general, children who went on to develop ASD had decreased urinary dimethylamine (estimate: −0.036; 95% CI: −0.065, −0.007), guanidoacetate (estimate: −0.109; 95% CI: −0.210, −0.007), hippurate (estimate: −0.136; 95% CI: −0.247, −0.025), and serine (estimate: −0.083; 95% CI: −0.155, −0.012) compared to children who were later diagnosed with TD, while children who went on to develop Non-TD had higher urinary ethanolamine (estimate: 0.074; 95% CI: 0.029, 0.118) and hypoxanthine (estimate = 0.098; 95% CI: 0.007, 0.188) but lower methionine (estimate: −0.085; 95% CI: −0.167, −0.002) and homovanillate (estimate = −0.085; 95% CI: −0.154, −0.016) compared to children who went on to have typical development. Urinary 3-aminoisobutyrate was similarly lower among children who were later diagnosed with ASD (estimate: −0.291; 95% CI: −0.429, −0.008) and Non-TD (estimate: −0.278; 95% CI: −0.486, −0.069) compared to controls. Effect size differences for metabolites of interest are presented in Figure 3.

**Figure 4:**
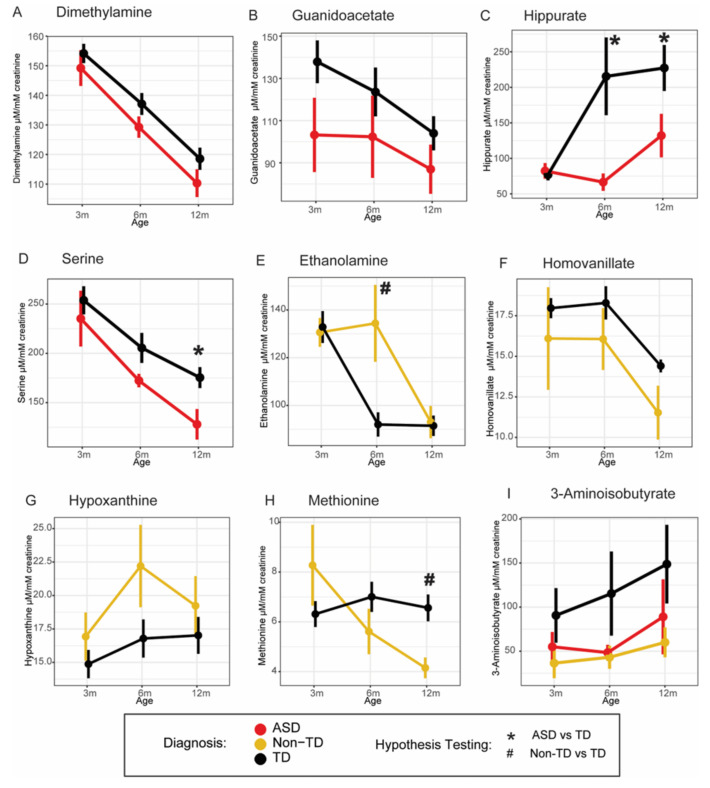
Comparison of urine metabolites that differed across time points in association with neurodevelopmental diagnosis based on GEE analysis. Urinary metabolites that differed among children who went on to develop ASD vs. TD: (**A**) dimethylamine, (**B**) guanidoacetate, (**C**) hippurate, and (**D**) serine. Metabolites that differed among children with Non-TD vs. TD: (**E**) Ethanolamine, (**F**) Homovanillate, (**G**) Hypoxanthine, (**H**) Methionine. (**I**) 3-Aminoisobutyrate differed for children diagnosed with ASD vs. Non-TD vs. TD. Data are presented as the mean ± SEM (*p* < 0.05 (unadjusted) as indicated).

Therefore, among our a priori hypothesized metabolites of one-carbon metabolism, children who went on to develop ASD tended to have lower urinary serine compared to TD controls. Additionally, we found some evidence that the gut-microbial co-metabolite hippurate was also lower among children who developed ASD compared to children who had typical neurodevelopment. Tryptophan, which has repeatedly been implicated in autism [12,15,16,17], also trended towards statistical significance (*p* < 0.10) with lower urinary concentrations among those who went on to develop ASD compared to neurotypical controls. These results may hint at the onset of metabolic shifts already occurring in the first year of life before neurodevelopmental diagnoses are made. For metabolites with *p*-values < 0.05 for GEE results, differences in metabolite concentration across time points and diagnosis are presented in Figure 4.

## 3. Discussion

This study aimed to investigate changes in the urinary metabolome to examine early biochemical markers associated with neurodevelopmental outcomes. Our results highlight subtle differences in several metabolites which differed among children who were later diagnosed with ASD and non-typical development (Non-TD) compared to typically developed (TD) controls in the first 12 months of life—years before neurodevelopmental assessments. Specifically, we found that urinary dimethylamine, guanidoacetate, and serine were all lower among children who went on to develop ASD compared to TD controls—of which, we had hypothesized a priori that serine and hippurate would differ among ASD cases. These decreased urinary metabolites had large effect size differences at 6 and 12 months, except for guanidinoacetate, which had large effect size differences at 3 and 6 months among ASD cases compared to controls. Children who went on to have Non-TD tended to have decreased methionine and homovanillate (both of which had large effect size differences at 3, 6, and 12 months) but elevated ethanolamine (which had a large effect size difference at 6 months) and hypoxanthine (with a large effect size difference at 3 and 6 months) compared to children who had typical neurodevelopment. We hypothesized a priori that methionine would differ among children with developmental disabilities though expected to see the difference among ASD cases rather than Non-TD cases. In addition, children who went on to develop ASD or Non-TD, similarly, had decreased urinary 3-aminoisobutyrate as compared to children who went on to have typical neurodevelopment. These results suggest that investigating the urinary metabolome in the first year of life may help characterize early metabolic shifts in key pathways associated with adverse neurodevelopmental and is worth exploring further.

For example, as previously discussed, one-carbon metabolism pathway abnormalities have repeatedly been implicated with autism. In the present study, we found that levels of urinary serine (an amino acid that plays a critical role as a methyl donor in the one-carbon metabolism folate cycle through its formation of S-adenosylmethionine (SAM)) tended to be lower among children who went on to develop ASD compared to children who did not. Others have also similarly found decreased urinary serine levels among children with ASD compared to controls [10,24,25]. While we had previously reported elevated plasma serine in ASD cases compared to age-matched controls [8], this discrepancy is likely due to the differences in biofluids analyzed (urine, a waste product, vs. plasma, which is maintained under tight homeostatic control) and/or due to variations in study participants (infants vs. children). In addition to its role in one-carbon metabolism, serine is also a precursor to other non-essential amino acids, the antioxidant glutathione, and plays a role in the synthesis of nucleotides. We also found that among children who went on to develop ASD or Non-TD, both tended to have decreased urinary 3-aminoisobutyrate (a catabolite of the nucleotide thymine). Ma et al. also found decreased urinary 3-aminoisobutyrate among ASD children (aged 2 to 18 years) compared to healthy controls. The methyl group of thymine is derived from a one-carbon intermediate originating from the interconversions of serine and glycine [26]. Therefore, decreased urinary 3-aminoisobutyrate may suggest diminished nucleotide metabolism conceivably due to altered serine levels. Interestingly, 3-aminoisobutyrate has been shown to improve insulin sensitivity and protect against high-fat-diet-induced obesity in mice [27]. Similarly, in a large human cohort study (*n* = 2067), plasma levels of 3-aminoisobutyrate were inversely correlated with plasma glucose, insulin, triglycerides, and total cholesterol, suggesting that 3-aminoisobutyrate may have beneficial metabolic properties. As such, decreased 3-aminoisobutyrate levels observed in our study may be related to decreased serine levels or may be related to adverse metabolic health (such as decreased insulin sensitivity, for example). Overall, alterations in serine metabolism may have extensive metabolic implications given the diverse roles of serine, including one-carbon metabolism, glutathione metabolism, and nucleic acid metabolism.

Another example of subtle metabolic changes which may already be arising in the first year of life are differences in amino acids involved in the production of major neurotransmitters. For instance, urinary tryptophan tended to be lower among children who went on to develop ASD compared to those who did not, though these trended towards significance (*p* < 0.09). However, dysregulated tryptophan metabolism has been proposed in the pathophysiology of autism, and numerous metabolomics analyses have also found altered tryptophan among individuals with ASD [12,15,16,17]. Tryptophan is an essential amino acid that serves as a biochemical precursor for serotonin, melatonin, and nicotinic acid (an important cofactor in metabolism). Yap et al. [23] previously found an increase in nicotinic acid metabolites among ASD cases compared to age-matched controls. The authors suggested this shift in tryptophan metabolism toward a shunt pathway resulted in increased formation of nicotinic acid and decreased production of the other tryptophan metabolites, such as those in the tryptophan–serotonin–melatonin pathways. This may explain why altered serotonin levels have previously been associated with mood disorders and temperament issues in children with ASD [28]. Furthermore, alterations in key regulatory enzymes, which compete for available tryptophan, have also been associated with autistic behaviors [29]. Interestingly, we also found decreased urinary homovanillate among children who went on to develop Non-TD compared to children with typical neurodevelopment. Homovanillate is the major terminal metabolite of the neurotransmitter dopamine and is excreted in urine when dopamine is broken down by the liver. Decreased dopamine has previously been associated with depression, schizophrenia, and autism [30]. Our results may indirectly suggest decreased levels of dopamine among children who went on to have Non-TD. Conversely, children who went on to develop Non-TD also had elevated urinary ethanolamine compared to children who went on to have typical development. Ethanolamine is a precursor of the excitatory neurotransmitter acetylcholine and is a major component of cell membranes as the phospholipid phosphoethanolamine [31]. An older study found that elevated urinary ethanolamine was associated with neuronal white matter degeneration and the authors suggested high ethanolamine in urine was derived from the increased breakdown of ethanolamine-containing phospholipids [32]. The elevated urinary ethanolamine we observed among children who were later diagnosed with Non-TD may indicate alterations in acetylcholine or phosphoethanolamine metabolism. Collectively, these results provide some evidence that key analytes involved with neurotransmitter-related pathways appear to be altered among children who ended up having neurodevelopmental disabilities.

We also found some evidence that children who went on to develop ASD tended to have lower urinary hippurate levels compared to children who went on to have typical neurodevelopment, pointing to differences in gut-microbial-related metabolites among individuals with ASD. Hippurate is a gut-microbial-host cometabolite primarily produced in the intestine by bacterial action on phenolic compounds of dietary origin [33]. While we did not directly investigate dietary differences among children who went on to develop ASD compared to TD (such as differences in consumption of phenolic-containing foods, such as fruits and vegetables, which may be common, even at an early age, among individuals with ASD), our models did adjust for age at introduction to solids to try to mitigate some possible dietary differences in our study population. Several other studies have found similar results among individuals with autism. Emond et al. [22] found decreased urinary hippurate concentrations in 6- to 9-year-old autistic children compared to age-matched healthy children, as did Nadal-Desbarats et al. who reported decreased urinary hippurate levels in thirty ASD children (ages 6–14) compared to healthy age-matched controls. Yap and colleagues [23] also found a trend for lower urinary hippurate levels in autistic children aged 3–9 years old compared to age-matched healthy controls and neurotypical siblings, although this was not statistically significant. In contrast, Lussu et al. [12] reported finding elevated urinary hippurate levels among Italian children with ASD (*n* = 21; ages 4–16 years) as compared to healthy siblings (*n* = 21; ages 4–17 years). Mussap et al. [34] also found elevated urinary hippurate among Italian children (ages 2–11 years old) among ASD cases (*n* = 31) compared to age-matched controls (*n* = 26). Kałużna-Czaplińska et al. also found elevated hippurate in the urine of autistic children (*n* = 35, ages 4–10 years) compared to non-autistic controls (*n* = 30, ages 4–10 years) [25]. Numerous factors which shape the gut microbiome (such as genetics, diet, lifestyle, ethnicity, and environment), may play a contributing role to the discrepancies observed among hippurate levels. Along the same line, we also found dimethylamine levels tended to be lower among children who went on to develop ASD compared to children who did not. Dimethylamine is derived from ingested choline and lecithin, and its formation is dependent on bacterial action in the intestine [35]. Similarly to hippurate, there are contradictory results in the literature regarding dimethylamine levels associated with autism. While decreased urinary dimethylamine was found among 6- to 14-year-old children with ASD, others reported elevated urinary dimethylamine among 3- to 9-year-old children with ASD [23]. Despite the discrepancies in levels of these gut-microbial cometabolites, taken together, these differences in hippurate and dimethylamine may suggest early differences in the gut microbiome specifically related to autism.

Notably, we did not find evidence of differences in the TCA cycle intermediates among children who went on to develop aberrant neurodevelopment, as hypothesized. However, it is possible that we did not see differences in TCA cycle metabolites as the kidneys, which are highly dependent on mitochondrial function [36], are still maturing, and the TCA cycle takes place in the mitochondrial matrix. At birth, the kidneys are considered immature and develop continuously until 6–12 months of age when the renal system becomes mature enough to concentrate urine like an adult. Additionally, exclusive breastfeeding vs. formula feeding, or the introduction of complementary food may play a contributing factor in the differences in the urinary metabolome during the first year of life. Indeed, there are clear differences in the urinary metabolome based on age in our PCA analysis (Figure 2) as indicated by shifts in the urinary metabolome, where samples at 3 months of age are more closely clustered together and begin to disperse with age. Additionally, the shifts we see in the first year of life are also likely due to the introduction of solid foods, which the American Academy of Pediatrics recommends introducing at approximately 6 months of age [37], although the average age of introduction of solid foods was around 5 months of age in our study population, with Non-TD cases slightly younger. Furthermore, parents of children with ASD frequently report that their children have selective eating behaviors and refuse many foods [38,39,40,41,42,43]. Feeding problems are estimated to affect 46% to 89% of children with ASD [42]. Although the great majority of studies investigating feeding issues in autism have been carried out in samples of children over the age of 3 years [42], some signs of feeding issues may arise before this age [44]. Food selectivity and pickiness can affect the urinary metabolome, as urine biomarkers may be correlated with habitual diet [45,46,47]. This is a limitation in our study, as we did not investigate the child’s diet in association with urinary metabolites, nor did we assess food problems in our present study.

A further limitation of this investigation is that our study participants are a subset of the MARBLES study—a high-risk ASD population with an older sibling with ASD. Study participants (even TD controls) were at elevated risk for ASD because of their family history of this condition. Therefore, our findings may not be generalizable to the greater ASD population as there may be a greater genetic contribution to the metabolic pathways discussed in the current study. Although the MARBLES study is relatively large (*n* = 260 at the time of our sample analysis), the current investigation was limited to individuals who had sufficient urine available for a metabolomics investigation collected at 3, 6, and/or 12 months of age, and therefore we had a relatively small sample size (*n* = 70). While the MARBLES study aims to collect urinary samples from all enrolled children during the first year of life on the day of the study visit, a clean-catch urine sample is not always available for each site visit. At that age, it is difficult to catch the infant when they are urinating; hence, very early postnatal samples were not always available. In the past, we attempted to use diapers with the idea that the urine could be extracted and analyzed, but after considerable effort trying this out, it was concluded that the matrix of the diaper would interfere with many analyte measurements (due to contaminants from the diapers, differences in diaper brands, differences in absorbency, possible fecal contamination, etc.). As such, we had a limited sample size available for the present investigation and were unable to successfully analyze samples at 3, 6, and 12 for all our study participants. A larger sample size may have better characterized early metabolic differences associated with adverse neurodevelopment. Furthermore, unlike the blood metabolome where analyte concentrations are narrowly maintained, urine concentrations can vary from sample to sample depending on hydration status (water reabsorption). However, to mitigate this, we normalized metabolites to urinary creatinine concentration to control for variations in urine flow rate. Another limitation in our analysis was that we did not know the exact composition of breastmilk vs. formula which made up the child’s diet and may also influence the urinary metabolome. However, most children were reported to have received some breastmilk past 12 months of age with no differences based on diagnosis, and we adjusted our models for age of introduction to solid foods, to try to minimize these effects. On the other hand, a strength of this study is that we measured metabolite changes very early on before neurodevelopmental diagnoses were made. Most other comparable studies have investigated older children already diagnosed with autism [8,10,16,23,48,49]. An exception is a recent metabolomics investigation that analyzed dried blood spots (DBS) of newborns who were later diagnosed with ASD (*n* = 37) compared to controls (*n* = 37) [50]. DBS are routinely collected shortly after birth and have the potential to identify biochemical markers of disease present at birth. Although that study was able to identify metabolites previously associated with ASD in DBS, none of these features remained significant after adjusting for FDR correction.

It is worth noting that there are several analytical platforms for carrying out metabolomics studies. The most common are NMR (as conducted in this analysis) and mass-spectrometry-based (MS) analysis—each brings its advantages and limitations. Unlike MS, NMR is inherently quantitative and requires little sample preparation. However, the sensitivity of NMR spectroscopy is a limitation compared to MS (which can be combined with different approaches, such as liquid chromatography (LC) and gas chromatography (GC), to increase the number of metabolites detected). For example, a previous GC-MS-based analysis was used to detect volatile organic compounds (VOCs) in the urine of 24 autistic children compared to 21 healthy controls [50]. Because of their volatility, structural diversity, and differences in polarity, urine VOCs are difficult to measure. However, in that study, researchers used solid-phase microextraction (SPME) coupled with GC-MS to successfully differentiate healthy controls from autistic cases based on VOC profiles. Other MS-based metabolomics analyses have also previously found abnormal tryptophan metabolism [16], altered TCA cycle [51], elevated concentrations of organic acids and sugars [15], differences in microbial co-metabolites [10,15,22,52], and altered amino acid metabolism [10,50] among children with ASD as compared to healthy controls, many of which indeed overlap with other NMR-based metabolomics analyses in ASD [7,13,23,48]. Given the dynamic range of metabolite concentrations and structural diversity within a biological sample, the use of complementary and comprehensive analytical cross-platform approaches, e.g., NMR, gas chromatography (GC)-MS, and liquid chromatography (LC)-MS, rather than a single technique may be utilized in future analysis to cope with analyte diversity.

Overall, our results highlight changes in one-carbon metabolism, gut-microbial co-metabolism, and neurotransmitter precursors that may be worth monitoring early in life, especially among children with an increased risk of autism, such as those with an older sibling with autism. Additionally, we did not find evidence of altered TCA cycle abnormalities at this age range. A larger sample size will be required to pinpoint early robust metabolites associated with ASD and other neurodevelopmental disorders. Further investigating early changes in metabolic pathways may provide better clues about what sort of biomedical and early intervention may help mitigate neurodevelopmental symptoms and severity.

## 4. Materials and Methods

### 4.1. Study Population

All study participants are a subset of the MARBLES (Markers of Autism Risk in Babies—Learning Early Signs) study [53]. The MARBLES study is an enriched-risk prospective cohort that follows pregnant women who are at high risk for delivering another infant(s) who will develop ASD, primarily because they previously delivered a child who developed ASD [54]. Although all these offspring are at high risk, only some (~20 %) will develop ASD, others will have different developmental outcomes, and many will develop typically. MARBLES began recruiting mothers in 2006. MARBLES families are primarily recruited from lists of children receiving services for autism through the California Department of Developmental Disabilities, as well as from other studies, by self- or provider referrals and obstetrics/gynecology clinics. Inclusion criteria of MARBLES are (1) the mother or father has a child or other first-degree relative with ASD; (2) the mother is 18 years old or older; (3) the mother is pregnant; (4) the mother speaks, reads, and understands English; and (5) mother resides within 2.5 h of the Davis/Sacramento region at the time of enrollment. For families who consent to participate in the MARBLES study, demographic information, medical records, outcomes, exposures, confounders, and biological specimens are all collected prospectively. The MARBLES study was approved by the State of California Department of Developmental Services and the institutional review board at the University of California Davis. Informed consent was obtained from all parents prior to enrollment. No data or specimens were collected or analyzed until informed consent was obtained. The informed consent included analysis of all specimens for any research related to child development. This study is reported in accordance with Strengthening the Reporting of Observational Studies in Epidemiology [55] (Supplementary Table S1).

At 36 months of age, children were assessed for ASD by a licensed clinical psychologist using the gold standard Autism Diagnostic Observation Schedules (ADOS) [56]. Cognitive development was assessed using the Mullen Scale of Early Learning (MSEL) with four subscales including visual reception, fine motor, receptive language, and expressive language. Neurodevelopmental outcomes were determined using both the ADOS and MSEL scores. Participants with ASD outcomes had scored over the ADOS cutoff and met the Diagnostic and Statistical Manual of Mental Disorder 5th edition (DSM-5) criteria for ASD. Participants with non-typical development (Non-TD) outcomes had scores within three points of the ADOS cutoff and/or Mullen Scores 1.5 to 2 standard deviations below average. The rest of the samples were classified as typical development. Details of the selection criteria for each categorization are available elsewhere [53].

For the present study, we investigated the urinary metabolome in association with neurodevelopmental diagnosis. In 02/2019, when we began to query samples available for the present analysis, 260 children who had completed the MARBLES study were considered for inclusion in this metabolomics analysis. However, 176 of these children were excluded as they did not have at least one clean-catch urine sample collected between 3 and 12 months of age available for the present investigation. Additionally, children missing a final neurodevelopmental diagnosis (*n* = 13) due to moving out of state or dropping from the study were also excluded. Furthermore, one urine sample was shown to have high levels of acetate, butyrate, and propionate and was removed from the analysis due to suspected fecal contamination [57]. Therefore, a total of 70 children (TD *n* = 42, ASD *n* = 17, Non-TD *n* = 11) with urine collected at 3, 6, and/or 12 months of age for a total of 109 spot urine samples were investigated in this analysis. Each participant contributed 1 to 3 urine samples for metabolomics analysis (Supplementary Tables S2 and S3).

### 4.2. ^1^H-NMR Metabolomics Analysis

Urinary biospecimens were collected using a pediatric urine bag and subsequently stored at −80 °C at the UC Davis biorepository. Urine samples for our study participants were collected from 03/2014 to 12/2018. ^1^H-NMR analysis was conducted from 04/2019 to 05/2019. For metabolomics analysis, urine samples were thawed and prepared by centrifuging to remove particulate matter, and 65 μL of internal standard (Chenomx Inc., Edmonton, AB, Canada) (consisting of ~5 mM DSS [sodium 2,2-dimethyl-2-silapentane-5-sulfonate, and 0.2% sodium azide in 99% D2O]) was added to 585 μL of supernatant, as described by Slupsky et al. [58]. The pH of each sample was adjusted to 6.8 ± 0.1 by the addition of small amounts of NaOH or HCl. The volumes of HCl and NaOH added were recorded. A 600 µL aliquot of the mixture was then transferred to a labeled 5 mm Bruker NMR tube and stored at 4 °C until NMR acquisition (within 24 h of sample preparation). Samples were run on a Bruker AVANCE 600 MHz NMR spectrometer equipped with a SampleJet autosampler using the NOESY-presaturation pulse sequence (noesypr). NMR spectra were acquired at 25 °C, with water saturation of 2.5 s during the prescan delay, a mixing time of 100 ms, 12 ppm sweep width, an acquisition time of 2.5 s, 8 dummy scans, and 32 transients. All spectra were zero-filled to 128 K data points and Fourier transformed with a 0.5-Hz line broadening applied. Spectra were manually phased and baseline-corrected and metabolites were identified and quantified using NMR Suite v8.1 (Chenomx Inc., Edmonton, AB, Canada) [59]. Subsequently, a list of compounds together with their respective concentrations, based on the concentration of the added internal standard (DSS-d6), was generated. All compounds in the database have been verified against known concentrations of reference NMR spectra of the pure compounds and are reproducible and accurate [58]. Investigators were blinded to child diagnosis and any participant information during sample preparation as well as NMR data acquisition and spectral analysis.

### 4.3. Statistical Analysis

Metabolite concentrations were expressed as micromole of metabolite per millimole of creatinine (/mmole creatinine) and log-transformed before analysis to approximate normality. Unsupervised principal component analysis (PCA) was used to identify inherent cluster detection and examine patterns in the metabolomic profiles. PCA was performed using the “prcomp” function, where each variable was centered by subtracting the variable means (center = True) but not scaled to the standard deviation (scale = FALSE) using ggplot2 library in R. Generalized estimating equation (GEE) analysis was performed on each metabolite to examine changes in metabolite concentrations in relation to adverse neurodevelopment. This method provides robust variance estimates, which account for the correlation among repeated observations in the same individuals and allows the characterization of effects from time-varying factors. GEE models were performed using the “Proc Genmod” function in SAS using a linear link and autoregressive correlation structure. Possible confounders were selected a priori based on a directed acyclic graph (DAG) (Supplementary Figure S1). The DAG was constructed using variables broadly associated (*p* < 0.20) with the neurodevelopmental diagnosis and urinary metabolites. Covariates considered in our DAG were the child’s sex, race/ethnicity, maternal age at the child’s birth, child’s age at introduction of solid foods, and attributes of maternal socioeconomic variables such as parental homeownership, insurance payer at delivery, and maximum maternal education. From the DAG, we then identified a sufficient set of adjustment factors that would remove confounding and minimize the estimated associations between the diagnostic group and metabolites—only the child’s sex and age at the introduction to first solids met these criteria. Additionally, as we were interested in evaluating our results in the context of our previous plasma metabolomics investigation [8], we also included the child’s race/ethnicity and parental homeownership in our GEE models. Therefore, the final GEE models were adjusted for the child’s sex (male, female), age of introduction to first solids (continuous (months)), child’s race/ethnicity (White, Hispanic, other), and parental homeownership (homeowner, renter) are presented in our analysis. The application of a false discovery rate (FDR) resulted in non-significant findings for all metabolite data. Therefore, unadjusted *p*-values < 0.05, in combination with large effect sizes were used to interpret the results. For metabolites with *p*-values < 0.05, the effect size between ASD vs. TD and Non-TD vs. TD at each time point was evaluated using Cliff’s delta (δ) statistic (cliff.delta function from the effsize package). Effect sizes were interpreted as follows: |δ| < 0.33, small; |δ| < 0.474, medium; and |δ| > 0.475, large effect size in metabolite concentration differences [60]. Differences in metabolite concentration at each time point across diagnosis were evaluated using independent t-tests or 2-way ANOVA followed by post hoc Tukey.

## Figures and Tables

**Table 1:** Characteristics of the study participants by neurodevelopmental diagnosis.

	Diagnosis			
N = 70	TD (N = 42)	ASD (N = 17)	Non-TD (N = 11)	*p*-Value ^a^
Child sex, n (%)				
Female	21 (50.00%)	02 (11.76%)	06 (54.55%)	0.016 *
Male	21 (50.00%)	15 (88.24%)	05 (45.45%)	
Child race/ethnicity, n (%)				
Non-Hispanic White	15 (35.71%)	07 (41.18%)	05 (45.45%)	0.55
Hispanic, any race	15 (35.71%)	04 (23.53%)	05 (45.45%)	
Other ^b^	12 (28.57%)	06 (35.29%)	01 (09.09%)	
Age (months) of introduction to complementary foods/solids, mean (SD)				
	5.42 (0.92)	5.39 (1.04)	4.78 (1.30)	0.19
Age (months) when breastfeeding stopped, mean (SD)				
	14.2 (9.38)	12.2 (8.99)	13.1 (9.84)	0.76
Maternal age at child’s birth (years), mean (SD)				
	35.11 (4.60)	34.41 (3.93)	34.55 (4.70)	0.77
Maternal education, n (%)				
Some college or less	20 (47.62%)	09 (52.94%)	07 (63.64%)	0.63
Bachelor’s or higher	22 (52.38%)	08 (47.06%)	04 (36.36%)	
Insurance delivery type ^c^, n (%)				
Private	34 (85.00%)	14 (82.35%)	08 (72.73%)	0.61
Public	06 (15.00%)	03 (17.65%)	03 (27.27%)	
Parental homeownership ^c^, n (%)				
Renter	14 (35.00%)	09 (56.25%)	04 (36.36%)	0.33
Homeowner	26 (65.00%)	07 (43.75%)	07 (63.64%)	

^a^ *p*-value from the Pearson’s chi-squared test for categorical variables and ANOVA test for continuous variables, * *p*-value < 0.05; ^b^ includes Black/African American (1%), Asian (17%), and multiracial (1%); ^c^ missing information (*n*): age (months) at the introduction to complementary foods/solids (3), insurance delivery type (2), homeownership (3).

**Table 2:** Changes (β) in metabolite concentrations of children with ASD and Non-TD compared to TD controls estimated by GEE analysis. Models were adjusted for the child’s sex, age of introduction to complementary foods, child’s race/ethnicity, and parental homeownership. *p*-values <0.05 (unadjusted) are shown in bold.

Class/Pathways	Metabolite ^1^	Dx	β	(95% CI)	*p*
Amino acid metabolism	1-Methylnicotinamide	ASD	−0.002	(−0.1161, 0.1112)	0.967
		Non-TD	0.098	(−0.0270, 0.2237)	0.124
	2-Oxobutyrate	ASD	−0.026	(−0.1103, 0.0577)	0.540
		Non-TD	0.075	(−0.0458, 0.1953)	0.225
	3-Aminoisobutyrate	ASD	−0.219	(−0.4293, −0.0085)	**0.041**
		Non-TD	−0.278	(−0.4866, −0.0698)	**0.009**
	3-Hydroxyisovalerate	ASD	−0.047	(−0.1462, 0.0521)	0.352
		Non-TD	−0.096	(−0.2133, 0.0221)	0.111
	3-Methylhistidine	ASD	−0.029	(−0.1085, 0.0506)	0.476
		Non-TD	−0.004	(−0.0961, 0.0887)	0.938
	4-Aminobutyrate	ASD	0.002	(−0.0767, 0.0798)	0.969
		Non-TD	−0.031	(−0.1270, 0.0645)	0.523
	Asparagine	ASD	0.016	(−0.0916, 0.1233)	0.773
		Non-TD	0.000	(−0.1078, 0.1084)	0.995
	Carnosine	ASD	−0.104	(−0.2706, 0.0637)	0.225
		Non-TD	−0.011	(−0.2039, 0.1824)	0.913
	Creatine	ASD	−0.213	(−0.5197, 0.0934)	0.173
		Non-TD	0.164	(−0.0916, 0.4185)	0.209
	Creatinine	ASD	−0.006	(−0.1383, 0.1258)	0.926
		Non-TD	−0.030	(−0.1717, 0.1109)	0.673
	Guanidoacetate	ASD	−0.109	(−0.2103, −0.007)	**0.036**
		Non-TD	−0.015	(−0.1049, 0.0753)	0.747
	Histidine	ASD	−0.078	(−0.2056, 0.0492)	0.229
		Non-TD	−0.008	(−0.1497, 0.1330)	0.908
	Lysine	ASD	−0.050	(−0.1868, 0.0869)	0.475
		Non-TD	−0.085	(−0.2245, 0.0553)	0.236
	Proline	ASD	−0.027	(−0.1184, 0.0651)	0.569
		Non-TD	0.007	(−0.0810, 0.0953)	0.874
	Taurine	ASD	−0.011	(−0.1662, 0.1436)	0.886
		Non-TD	0.010	(−0.1274, 0.1481)	0.883
	Threonine	ASD	0.022	(−0.0729, 0.1175)	0.647
		Non-TD	0.090	(−0.0200, 0.1990)	0.109
	Urocanate	ASD	0.027	(−0.0630, 0.1168)	0.558
		Non-TD	0.069	(−0.0402, 0.1772)	0.217
Branched chain amino acid metabolism, amino acid metabolism	3-Hydroxy−3-methylglutarate	ASD	−0.006	(−0.0550, 0.0430)	0.809
		Non-TD	−0.002	(−0.0519, 0.0489)	0.954
	3-Hydroxyisobutyrate	ASD	−0.007	(−0.0905, 0.0767)	0.871
		Non-TD	0.042	(−0.0409, 0.1256)	0.319
	Isoleucine	ASD	−0.012	(−0.0909, 0.0665)	0.761
		Non-TD	0.030	(−0.0467, 0.1065)	0.444
	Leucine	ASD	−0.009	(−0.0968, 0.0791)	0.843
		Non-TD	−0.010	(−0.1155, 0.0952)	0.850
	Valine	ASD	0.012	(−0.0586, 0.0830)	0.736
		Non-TD	0.001	(−0.0806, 0.0835)	0.973
Glutathione metabolism	2-Aminobutyrate	ASD	−0.055	(−0.1982, 0.0887)	0.455
		Non-TD	−0.030	(−0.1866, 0.1257)	0.702
Glutathione metabolism, amino acid metabolism	2-Hydroxybutyrate	ASD	−0.023	(−0.1304, 0.0842)	0.673
		Non-TD	0.003	(−0.0922, 0.0973)	0.958
Glycine, serine, and threonine metabolism, homocysteine metabolism, lipid metabolism	Choline	ASD	0.010	(−0.1479, 0.1671)	0.905
		Non-TD	−0.025	(−0.0991, 0.0500)	0.518
Glycine, serine, and threonine metabolism, one-carbon metabolism, amino acid metabolism	Serine	ASD	−0.083	(−0.1549, −0.0117)	**0.023**
		Non-TD	−0.058	(−0.1282, 0.0132)	0.111
Glycine, serine, and threonine metabolism, homocysteine metabolism	Betaine	ASD	0.067	(−0.0519, 0.1863)	0.269
		Non-TD	−0.052	(−0.2141, 0.1100)	0.529
	N,N-Dimethylglycine	ASD	−0.013	(−0.1341, 0.1089)	0.839
		Non-TD	−0.006	(−0.1964, 0.1845)	0.951
Glycine, serine, and threonine metabolism, homocysteine metabolism, glutathione metabolism, amino acid metabolism	Glycine	ASD	−0.013	(−0.1448, 0.1195)	0.851
		Non-TD	−0.020	(−0.1132, 0.0727)	0.669
Glycolysis	Lactate	ASD	−0.002	(−0.1137, 0.1094)	0.970
		Non-TD	0.022	(−0.0999, 0.1446)	0.720
	Pyruvate	ASD	−0.012	(−0.1355, 0.1111)	0.847
		Non-TD	0.047	(−0.0546, 0.1489)	0.364
Glycolysis, amino acid metabolism	Alanine	ASD	−0.024	(−0.1294, 0.0819)	0.660
		Non-TD	−0.016	(−0.1151, 0.0833)	0.754
Homocysteine metabolism, methionine cycle, amino acid metabolism	Methionine	ASD	−0.050	(−0.1683, 0.0677)	0.403
		Non-TD	−0.085	(−0.1672, −0.0022)	**0.044**
Ketone bodies	3-Hydroxybutyrate	ASD	−0.120	(−0.2594, 0.0191)	0.091
		Non-TD	−0.062	(−0.1690, 0.0446)	0.254
	Acetoacetate	ASD	0.014	(−0.1191, 0.1468)	0.839
		Non-TD	0.034	(−0.1020, 0.1703)	0.623
	Acetone	ASD	0.027	(−0.1237, 0.1776)	0.726
		Non-TD	0.034	(−0.1707, 0.2388)	0.745
Lipid-related metabolism	Carnitine	ASD	0.174	(−0.0425, 0.3903)	0.115
		Non-TD	0.014	(−0.2345, 0.2622)	0.913
	O-Acetylcarnitine	ASD	0.069	(−0.1149, 0.2537)	0.461
		Non-TD	−0.026	(−0.1911, 0.1398)	0.761
Neurotransmitter precursor amino acid, amino acid metabolism	Phenylalanine	ASD	−0.013	(−0.1189, 0.0937)	0.816
		Non-TD	−0.001	(−0.0777, 0.0762)	0.985
	Tyrosine	ASD	−0.064	(−0.1563, 0.0274)	0.169
		Non-TD	0.002	(−0.0971, 0.1011)	0.969
Neurotransmitter precursor amino acid, glutathione metabolism, amino acid metabolism	Glutamate	ASD	0.025	(−0.0826, 0.1334)	0.645
		Non-TD	−0.006	(−0.0844, 0.0719)	0.876
	Glutamine	ASD	−0.012	(−0.0905, 0.0662)	0.761
		Non-TD	−0.013	(−0.1067, 0.0811)	0.790
Other	2-Hydroxyisobutyrate	ASD	0.020	(−0.0731, 0.1129)	0.676
		Non-TD	−0.041	(−0.1179, 0.0356)	0.293
	4-Hydroxyphenylacetate	ASD	0.068	(−0.0641, 0.1991)	0.315
		Non-TD	−0.090	(−0.2625, 0.0836)	0.311
	Adipate	ASD	0.002	(−0.1411, 0.1444)	0.982
		Non-TD	0.076	(−0.0379, 0.1905)	0.190
	Ascorbate	ASD	−0.111	(−0.4801, 0.2583)	0.556
		Non-TD	0.026	(−0.3430, 0.3942)	0.892
	Dimethylamine	ASD	−0.036	(−0.0646, −0.0072)	**0.014**
		Non-TD	−0.020	(−0.0490, 0.0088)	0.173
	Ethanolamine	ASD	−0.025	(−0.1043, 0.0543)	0.537
		Non-TD	0.074	(0.0291, 0.1184)	**0.001**
	Ethylmalonate	ASD	−0.001	(−0.1267, 0.1245)	0.987
		Non-TD	−0.070	(−0.2024, 0.0619)	0.298
	Formate	ASD	0.143	(−0.0232, 0.3082)	0.092
		Non-TD	−0.132	(−0.2644, 0.0004)	0.051
	Glycolate	ASD	−0.024	(−0.1259, 0.0785)	0.649
		Non-TD	−0.004	(−0.1516, 0.1443)	0.962
	Homovanillate	ASD	−0.004	(−0.0560, 0.0476)	0.873
		Non-TD	−0.085	(−0.1539, −0.0156)	**0.016**
	Hypoxanthine	ASD	−0.009	(−0.0954, 0.0765)	0.830
		Non-TD	0.098	(0.0076, 0.1883)	**0.034**
	Methanol	ASD	0.049	(−0.0706, 0.1689)	0.421
		Non-TD	0.011	(−0.1641, 0.1854)	0.905
	Methylguanidine	ASD	−0.001	(−0.0690, 0.0666)	0.972
		Non-TD	−0.035	(−0.0803, 0.0106)	0.132
	Pantothenate	ASD	0.049	(−0.0715, 0.1700)	0.424
		Non-TD	0.002	(−0.0907, 0.0941)	0.972
	Quinolinate	ASD	−0.044	(−0.1296, 0.0423)	0.320
		Non-TD	0.004	(−0.1313, 0.1389)	0.956
	Trigonelline	ASD	−0.054	(−0.1897, 0.0813)	0.433
		Non-TD	0.029	(−0.1421, 0.2000)	0.740
	Uracil	ASD	−0.049	(−0.1545, 0.0564)	0.362
		Non-TD	0.034	(−0.0464, 0.1146)	0.407
	Urea	ASD	−0.010	(−0.0933, 0.0739)	0.820
		Non-TD	−0.011	(−0.1232, 0.1009)	0.846
	Xanthosine	ASD	−0.012	(−0.1087, 0.0846)	0.807
		Non-TD	−0.025	(−0.1152, 0.0644)	0.579
Other, bacterial metabolite	1,2-Propanediol	ASD	0.147	(−0.2108, 0.5039)	0.422
		Non-TD	−0.111	(−0.4332, 0.2108)	0.498
	Dimethyl sulfone	ASD	0.027	(−0.1084, 0.1631)	0.693
		Non-TD	0.079	(−0.0477, 0.2060)	0.222
	Hippurate	ASD	−0.136	(−0.2474, −0.0248)	**0.017**
		Non-TD	−0.110	(−0.3162, 0.0954)	0.293
	Trimethylamine	ASD	−0.007	(−0.0764, 0.0628)	0.848
		Non-TD	−0.060	(−0.1197, 0.0007)	0.053
	Trimethylamine-N-oxide	ASD	0.023	(−0.2011, 0.2465)	0.843
		Non-TD	0.112	(−0.1495, 0.3740)	0.401
Short-chain fatty acids	Acetate	ASD	0.089	(−0.1046, 0.2815)	0.369
		Non-TD	−0.014	(−0.2219, 0.1949)	0.899
	Butyrate	ASD	0.033	(−0.0865, 0.1519)	0.591
		Non-TD	−0.011	(−0.1153, 0.0942)	0.843
	Propionate	ASD	0.017	(−0.1438, 0.1779)	0.835
		Non-TD	−0.060	(−0.2233, 0.1033)	0.472
Sugars and their derivatives	Fucose	ASD	−0.077	(−0.2306, 0.0764)	0.325
		Non-TD	−0.039	(−0.1815, 0.1028)	0.588
	Galactose	ASD	−0.054	(−0.3535, 0.2454)	0.724
		Non-TD	−0.031	(−0.3001, 0.2390)	0.824
	Gluconate	ASD	0.035	(−0.0717, 0.1422)	0.519
		Non-TD	0.068	(−0.0340, 0.1697)	0.192
	Glucose	ASD	−0.070	(−0.1782, 0.0379)	0.203
		Non-TD	−0.030	(−0.1106, 0.0513)	0.473
	Lactose	ASD	0.014	(−0.1285, 0.1561)	0.849
		Non-TD	0.041	(−0.1356, 0.2169)	0.652
	Mannitol	ASD	0.021	(−0.0686, 0.1113)	0.641
		Non-TD	0.028	(−0.0675, 0.1240)	0.563
	myo Inositol	ASD	0.093	(−0.0580, 0.2432)	0.228
		Non-TD	0.035	(−0.1126, 0.1834)	0.639
Tricarboxylic acid cycle	2-Oxoglutarate	ASD	−0.039	(−0.2292, 0.1507)	0.686
		Non-TD	0.135	(−0.0973, 0.3663)	0.255
	cis Aconitate	ASD	0.026	(−0.0927, 0.1445)	0.668
		Non-TD	0.043	(−0.0386, 0.1250)	0.301
	Citrate	ASD	0.036	(−0.0889, 0.1613)	0.571
		Non-TD	0.054	(−0.1154, 0.2234)	0.532
	Fumarate	ASD	−0.061	(−0.2150, 0.0926)	0.435
		Non-TD	0.048	(−0.1070, 0.2029)	0.544
	Succinate	ASD	0.010	(−0.1787, 0.1992)	0.915
		Non-TD	−0.049	(−0.2232, 0.1251)	0.581
Tryptophan metabolism, amino acid metabolism	3-Indoxylsulfate	ASD	−0.091	(−0.2464, 0.0645)	0.252
		Non-TD	−0.024	(−0.1817, 0.1338)	0.766
	Tryptophan	ASD	−0.072	(−0.145, 0.0019)	0.056
		Non-TD	−0.020	(−0.1027, 0.0627)	0.636

^1^ Metabolite concentrations are expressed as micromoles of metabolite per millimole of creatinine and were log-10 transformed before analysis.

## Data Availability

The data presented in this study are available on request from the corresponding author. The data are not publicly available due to the need to protect participant privacy.

## Supplementary Materials

The supporting information can be downloaded at: https://www.mdpi.com/article/10.3390/ijms24119454/s1.
