# Supplementary material for: Investigating the Urinary Metabolome in the First Year of Life and Its Association with Later Diagnosis of Autism Spectrum Disorder or Non-Typical Neurodevelopment in the MARBLES Study
Source: Int J Mol Sci. 2023 May 29;24(11):9454. doi: 10.3390/ijms24119454 (PMC10254021; doi:10.3390/ijms24119454)
Supplement: Supplementary file 1 [file ijms-24-09454-s001.zip › ijms-2408258-supplementary.pdf]

Supplementary Materials

Investigating the Urinary Metabolome in the First Year of Life and its Association with Later Diagnosis of Autism Spectrum Disorder or Non-Typical Neurodevelopment in the MARBLES Study

Authors

Jennie Sotelo-Orozco [1,*], Rebecca J. Schmidt [1,2], Carolyn M. Slupsky [3,4] and Irva Hertz-Picciotto [1,2]

[1] Department of Public Health Sciences, School of Medicine, University of California Davis, Davis, CA 95616, USA; rjschmidt@ucdavis.edu (R.J.S.); iher@ucdavis.edu (I.H.-P.)
[2] Medical Investigation of Neurodevelopmental Disorders (MIND) Institute, School of Medicine, University of California Davis, Sacramento, CA 95817, USA
[3] Department of Nutrition, University of California, Davis, CA 95616, USA; cslupsky@ucdavis.edu
[4] Department of Food Science and Technology, University of California, Davis, CA 95616, USA
* Correspondence: jssotelo@ucdavis.edu

Figures

Figure S1: Directed acyclic graph

Tables

Table S1: Strengthening the Reporting of Observational Studies in Epidemiology (STROBE) Checklist

Table S2: Total number of samples analyzed at each time point by diagnosis

Table S3: Possible combination and number of urine samples analyzed at 3, 6, and/or 12 months

Supplementary Figure S1: Directed acyclic graph (DAG) utilized to identify potential confounders. Green circles represent ancestors of the exposure, blue circles ancestors of the outcome, and pink circles ancestors of both exposure and outcome.

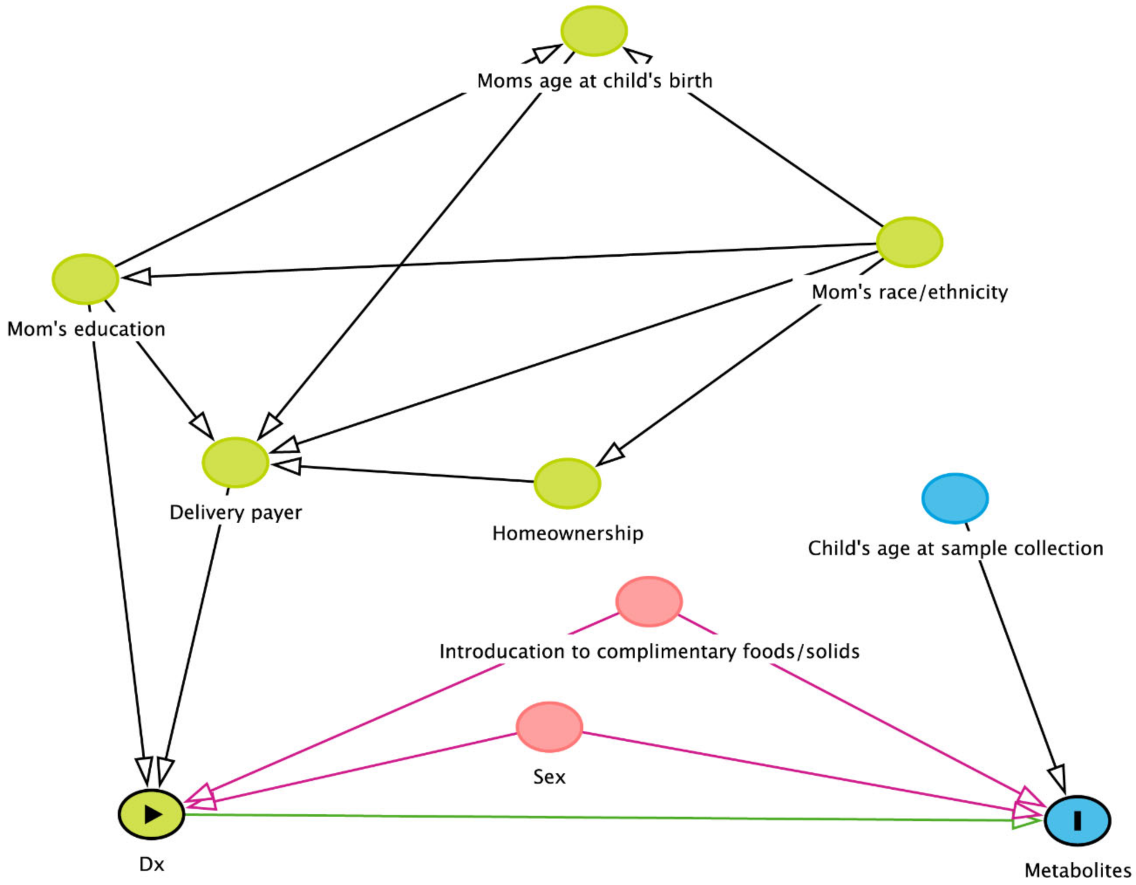

Supplementary Table S1: STROBE Statement—Checklist of items that should be included in reports of ***cohort studies***

	Item No	Recommendation	Page No.	Relevant text from manuscript
**Title and abstract**	1	(*a*) Indicate the study's design with a commonly used term in the title or the abstract	1	This is included in the abstract:<br>"This study included a subset of children (n= 70) from the Markers of Autism Risks in Babies –Learning Early Signs (MARBLES) prospective cohort study."
		(*b*) Provide in the abstract an informative and balanced summary of what was done and what was found	1	This information is stated in the study abstract (study objective described, methods and results described).
**Introduction**				
Background/rationale	2	Explain the scientific background and rationale for the investigation being reported	1-2	Rationale and existing literature are stated in the introduction.
Objectives	3	State specific objectives, including any prespecified hypotheses	2	"The objective of the present study was to investigate early alterations in key metabolic pathways by investigating differences in the urinary metabolome of infants at 3, 6, and 12 months of age in children who were later diagnosed with ASD, or non-typical development (Non-TD), as compared to neurotypical controls (TD)..."<br><br>"Building off the existing literature and our previous analysis, we hypothesized a priori that alterations in one-carbon metabolism-related metabolites (serine, betaine, n,n-dimethylglycine, glycine, choline, methionine, 2-aminobutyrate, and 2-hydroxybutyrate) would differ among children with ASD as compared to TD controls….."
**Methods**				
Study design	4	Present key elements of study design early in the paper	15-17	Study design is stated in the first subsection of Methods section. Key elements are all described in the methods.<br><br>"The MARBLES study is an enriched-risk prospective cohort that follows pregnant women who are at high risk for delivering another infant(s) who will develop ASD,

				primarily because they previously delivered a child who developed ASD."<br><br>"For the present study, we investigated the urinary metabolome in association with neurodevelopmental diagnosis.
Setting	5	Describe the setting, locations, and relevant dates, including periods of recruitment, exposure, follow-up, and data collection	15-16	Location of study area, dates of sample collection and analysis are described in the methods section under "Study Population" and "^{1}H-NMR Metabolomics analysis"
Participants	6	(*a*) Give the eligibility criteria, and the sources and methods of selection of participants. Describe methods of follow-up	15-16	Study population is described in the method section as well as selection criteria.<br><br>"Inclusion criteria of MARBLES are 1) the mother or father has a child or other first-degree relative with ASD; 2) the mother is 18 years old or older; 3) the mother is pregnant; 4) the mother speaks, reads, and understands English; and 5) mother resides within 2.5 h of the Davis/Sacramento region at the time of enrollment"
		(*b*) For matched studies, give matching criteria and number of exposed and unexposed		N/A
Variables	7	Clearly define all outcomes, exposures, predictors, potential confounders, and effect modifiers. Give diagnostic criteria, if applicable	15-17	Diagnostic criteria is explained in methods section under "Study Population".<br><br>"At 36 months of age, children were assessed for ASD by a licensed clinical psychologist using the gold standard Autism Diagnostic Observation Schedules (ADOS) [50]. Cognitive development was assessed using the Mullen Scale of Early Learning (MSEL) with four subscales including visual reception, fine motor, receptive language, and expressive language. Neurodevelopmental outcomes were determined using both the ADOS and MSEL scores. Participants with ASD outcomes had scored over the ADOS cutoff and met the Diagnostic and Statistical Manual of Mental Disorder 5th edition (DSM-5) criteria for ASD. Participants with

				non-typical development (Non-TD) outcomes had scores within three points of the ADOS cutoff and/or Mullen Scores 1.5 to 2 standard deviations below average. The rest of the samples were classified as typical development."<br><br>Potential confounders are described in methods section under "Statistical analysis". "Possible confounders were selected a priori based on a directed acyclic graph (DAG) (Supplementary Figure S1). The DAG was constructed using variables broadly associated (P < 0.20) with the neurodevelopmental diagnosis and urinary metabolites. Covariates considered in our DAG were the child's sex, race/ethnicity, maternal age at the child's birth, child's age at introduction of solid foods, and attributes of maternal socioeconomic variables such as parental homeownership, insurance payer at delivery, and maximum maternal education.:
Data sources/ measurement	8*	For each variable of interest, give sources of data and details of methods of assessment (measurement). Describe comparability of assessment methods if there is more than one group	15-17	Data collection and sample collections are described in methods section under "study population" and "1H-NMR metabolomics analysis".
Bias	9	Describe any efforts to address potential sources of bias	15-17	We notably tried to reduce bias by excluding samples without known neurodevelopmental diagnosis, and possibly contaminated samples. "Additionally, children missing a final neurodevelopmental diagnosis (n=13) were excluded. Furthermore, one urine sample was shown to have high levels of acetate, butyrate, and propionate and was removed from analysis due to suspected fecal contamination."<br><br>We also attempted to reduce confounding effects by adjusting our models for possible confounders. "From the DAG, we then identified a sufficient set of adjustment factors that would remove confounding and minimize the estimated associations between the diagnostic group and metabolites— only the child's sex and

				age at the introduction to first solids met these criteria.”
Study size	10	Explain how the study size was arrived at	16	Our study size is explained in our methods “study population” section. “For the present study, we investigated the urinary metabolome in association with neurodevelopmental diagnosis. In 02/2019, when we began to query samples available for the present analysis, 260 MARBLES children who had completed the MARBLES study were considered for inclusion in this metabolomics analysis. However, 176 of these children were excluded as they did not have at least one clean-catch urine sample collected between 3-12 months of age available for the present investigation. Additionally, children missing a final neurodevelopmental diagnosis (n=13) due to moving out of state or dropping from the study were also excluded. Furthermore, one urine sample was shown to have high levels of acetate, butyrate, and propionate and was removed from analysis due to suspected fecal contamination. Therefore, a total of 70 children (TD n=42, ASD n=17, Non-TD n=11) with urine collected at 3, 6, and/or 12 months of age for a total of 109 spot urine samples were investigated in this analysis.”
Quantitative variables	11	Explain how quantitative variables were handled in the analyses. If applicable, describe which groupings were chosen and why	15-17	Quantitative metabolite data are described in methods “statistical analysis” section.<br>“Metabolite concentrations were expressed as micromole of metabolite per milli-mole of creatinine (/mmole creatinine), and log-transformed before analysis to approximate normality.”<br><br>Definitions for categorical variables are described in the methods “statistical analysis” section.<br>“Therefore, the final GEE models were adjusted for the child’s sex (male, female), age of introduction to first solids (continuous (months)) child’s race/ethnicity (white, Hispanic, other), and parental homeownership (homeowner, renter) are presented in our analysis”
Statistical methods	12	(*a*) Describe all statistical methods, including those	17	This is described in detail in the methods “statistical analysis” section.

		used to control for confounding		"Unsupervised principal component analysis (PCA) was used to identify inherent cluster detection and examine patterns in the metabolomic profiles. PCA was performed using the "prcomp" function where each variable was centered by subtracting the variable means (center=True) but not scaled to the standard deviation (scale=FALSE) using ggplot2 library in R. Generalized estimating equation (GEE) analysis was per-formed on each metabolite to examine longitudinal changes in metabolite concentrations in relation to adverse neurodevelopment….. Possible confounders were selected a priori based on a directed acyclic graph (DAG) (Supplementary Figure S1). The DAG was constructed using variables broadly associated (P < 0.20) with the neurodevelopmental diagnosis and urinary metabolites. Covariates considered in our DAG were the child's sex, race/ethnicity, maternal age at the child's birth, child's age at introduction of solid foods, and attributes of maternal socioeconomic variables such as parental homeownership, insurance payer at delivery, and maximum maternal education. From the DAG, we then identified a sufficient set of adjustment factors that would remove confounding and minimize the estimated associations between the diagnostic group and metabolites— only the child's sex and age at the introduction to first solids met these criteria. Additionally, as we were interested in evaluating our results in the context of our previous plasma metabolomics investigation, we also included the child's race/ethnicity and parental homeownership in our GEE models."
		(*b*) Describe any methods used to examine subgroups and interactions	17	N/A
		(*c*) Explain how missing data were addressed	16	Samples with missing data were excluded. "260 children who had completed the MARBLES study were considered for inclusion for this analysis, however, 176 were excluded as they did not have at least one urine sample collected between 3-12 months of age available for metabolomics investigation. Additionally, children missing a final

				neurodevelopmental diagnosis (n=13) were excluded.”
		(*d*) If applicable, explain how loss to follow-up was addressed		N/A
		(*e*) Describe any sensitivity analyses		N/A
**Results**				
Participants	13*	(a) Report numbers of individuals at each stage of study—eg numbers potentially eligible, examined for eligibility, confirmed eligible, included in the study, completing follow-up, and analysed	15-16, 4	This is described in the methods “Study participants” section, as well as presented in Figure 1.
		(b) Give reasons for non-participation at each stage	4	Exclusion at each stage is presented in figure 1.
		(c) Consider use of a flow diagram	4	Figure 1 presents a flow chart of study population.
Descriptive data	14*	(a) Give characteristics of study participants (eg demographic, clinical, social) and information on exposures and potential confounders	3	Characteristics of study participants are fully presented in Table 1.
		(b) Indicate number of participants with missing data for each variable of interest	3	Any missing data is indicated in the footnote of Table 1. “Missing information (n): Age (months) at the introduction to complementary foods/ solids (3), insurance delivery type (2), homeownership (3)”
		(c) Summarise follow-up time (eg, average and total amount)		N/A
Outcome data	15*	Report numbers of outcome events or summary measures over time	3, 7-10	All numbers are reported in Tables.
Main results	16	(*a*) Give unadjusted estimates and, if applicable, confounder-adjusted estimates and their precision (eg, 95% confidence interval). Make clear which confounders were adjusted for and why they were included	7-10	All adjusted estimates, and 95% confidence intervals, are reported in Table 2. Non-adjusted estimates were not displayed in the interest of table clarity, and due to the number of metabolites presented.

		(*b*) Report category boundaries when continuous variables were categorized	3	Category boundaries are presented in Table 1.
		(*c*) If relevant, consider translating estimates of relative risk into absolute risk for a meaningful time period		N/A
Other analyses	17	Report other analyses done—eg analyses of subgroups and interactions, and sensitivity analyses		A comparison of the urinary metabolites (present study) vs plasma metabolites analysis (previous study) is presented in Supplementary Table S3.
**Discussion**				
Key results	18	Summarise key results with reference to study objectives	11	This is presented in the first paragraph of the discussion section.
Limitations	19	Discuss limitations of the study, taking into account sources of potential bias or imprecision. Discuss both direction and magnitude of any potential bias	14	Limitations of the study are discussed in the discussion section.
Interpretation	20	Give a cautious overall interpretation of results considering objectives, limitations, multiplicity of analyses, results from similar studies, and other relevant evidence	11-15	References were added where possible and discussed. Limitations were taken into account in the discussion.
Generalisability	21	Discuss the generalisability (external validity) of the study results	14	We discuss the generalizability of the study results in the limitations section of our discussion section. "A further limitation of this investigation is that our study participants are a subset of the MARBLES study—a high-risk ASD population with an older sibling with ASD. Study participants (even TD controls) were at elevated risk for ASD because of their family history of this condition. Therefore, our findings may not be generalizable to the greater ASD population as there may be a greater genetic contribution to the metabolic pathways discussed in the current study."
**Other information**				
Funding	22	Give the source of funding and the role of the funders for the present study and, if applicable, for the original study on which the present article is based	18	Details of funding sources is presented in "Funding" section.

Supplementary Table S2:Total number of samples analyzed at each time point by diagnosis.

**Total samples analyzed at each time point (months)**	***Diagnosis***		
	TD	***ASD***	***Non-TD***
3	25	8	3
6	19	9	6
12	24	8	7

Supplementary Table S3: Possible combination and number of urine samples analyzed at 3, 6, and/or 12 months of age for the 70 children included in this analysis (TD, n=42; ASD, n=17; Non-TD, n=11). Each study participant contributed 1 to 3 urine samples at different time points. For example, five children with TD, one with ASD, and one child with Non-TD contributed samples at 3, 6, and 12 months of age (bottom row).

**Combinations of urine samples analyzed at each time point (months)**	***Diagnosis***		
	TD	***ASD***	***Non-TD***
3	10	4	1
6	4	3	2
12	7	3	4
3,6	4	2	1
3,12	6	1	0
6,12	6	3	2
3,6,12	5	1	1
